# The ABC of Pectus Excavatum: a novel anatomical classification system

**DOI:** 10.1186/1749-8090-10-S1-A277

**Published:** 2015-12-16

**Authors:** K Mazhar, I Cliff, N Watson, CMR Satur

**Affiliations:** 1 The Heart Centre, Royal Stoke University Hospital, Stoke-on-Trent, Staffordshire, ST46QG, UK

## Background/Introduction

Pectus Excavatum affects between 1:400 - 1000 live births. Little academic focus has been directed to the anatomical subgroups of this condition. No anatomical classification and subgrouping has been formulated. Current surgical strategies utilize a 'one size fits all' philosophy with variations of the Nuss or Ravitch procedure

## Aims/Objectives

Our aim was to stratify patients with Pectus Excavatum by providing an anatomical classification that would guide treatment. The novel classification we propose provides a framework for use with current and evolving medical technologies

## Method

Between April 2006 - March 2015, 24 patients (22 male : 2 female; Age 15 - 33 years) presented to our institute with symptomatic Pectus Excavatum. All presented with symptoms of dyspnoea /fatigue/dysphagia. All patients underwent Prospective evaluation of exercise and anatomical characteristics by: Spirometry; Cardiopulmonary exercise tolerance (CPEX) testing & Non-contrast axial/sagittal CT scan of the thorax & 3D reconstruction. We propose an Anatomical classification based on 2D and 3D CT imaging: Classification A : No depression of manubrium, posterior angulation of sternal body Classification B: (1) Horizontal depression of manubrium and sternal body with no angulation (2) Depression of manubrium with posterior angulation of sternal body Classification C: Complex asymmetrical torsion of manubrium and body of sternum associated with asymmetrical distortion and depression of rib cage

## Results

9 patients (38%) had a Type A defect. We recommend this sternal body angulation to be corrected with Ravitch Procedure, Transverse osteotomy and midline screw fixation bar. 14 (58%) patients had a Type B2 defect, which we recommend should be repaired as a Type A defect but elevation of the whole sternum using a transverse screw fixation bar.

## Discussion/Conclusion

Our classification provides a framework for evaluation of the pathological anatomy of Pectus Excavatum. This classification has been shown to have utilitarian value in planning surgical strategy. The model will provide opportunities for future development of surgical technique and provides a descriptor that will allow academic discussion

